# Alterations in Life-History Associated With Non-target-site Herbicide Resistance in *Alopecurus myosuroides*

**DOI:** 10.3389/fpls.2019.00837

**Published:** 2019-06-26

**Authors:** David Comont, Craig Knight, Laura Crook, Richard Hull, Roland Beffa, Paul Neve

**Affiliations:** ^1^Department of Biointeractions and Crop Protection, Rothamsted Research, Harpenden, United Kingdom; ^2^School of Life Sciences, University of Warwick, Wellesbourne, United Kingdom; ^3^Bayer AG, CropScience Division, Frankfurt, Germany

**Keywords:** non-target site resistance, resistance cost, fitness, life-history, trade-offs

## Abstract

The evolution of resistance to herbicides is a classic example of rapid contemporary adaptation in the face of a novel environmental stress. Evolutionary theory predicts that selection for resistance will be accompanied by fitness trade-offs in environments where the stress is absent. *Alopecurus myosuroides*, an autumn-germinating grass weed of cereal crops in North-West Europe, has evolved resistance to seven herbicide modes-of-action, making this an ideal species to examine the presence and magnitudes of such fitness costs. Here, we use two contrasting *A. myosuroides* phenotypes derived from a common genetic background, one with enhanced metabolism resistance to a commercial formulation of the sulfonylurea (ALS) actives mesosulfuron and iodosulfuron, and the other with susceptibility to these actives (S). Comparisons of plant establishment, growth, and reproductive potential were made under conditions of intraspecific competition, interspecific competition with wheat, and over a gradient of nitrogen deprivation. Herbicide dose response assays confirmed that the two lines had contrasting resistance phenotypes, with a 20-fold difference in resistance between them. Pleiotropic effects of resistance were observed during plant development, with R plants having a greater intraspecific competitive effect and longer tiller lengths than S plants during vegetative growth, but with S plants allocating proportionally more biomass to reproductive tissues during flowering. Direct evidence of a reproductive cost of resistance was evident in the nitrogen deprivation experiment with R plants producing 27% fewer seed heads per plant, and a corresponding 23% reduction in total seed head length. However, these direct effects of resistance on fecundity were not consistent across experiments. Our results demonstrate that a resistance phenotype based on enhanced herbicide metabolism has pleiotropic impacts on plant growth, development and resource partitioning but does not support the hypothesis that resistance is associated with a consistent reproductive fitness cost in this species. Given the continued difficulties associated with unequivocally detecting costs of herbicide resistance, we advocate future studies that adopt classical evolutionary quantitative genetics approaches to determine genetic correlations between resistance and fitness-related plant life history traits.

## Introduction

Plant evolutionary theory predicts that adaptations conveying resistance to an environmental stress can result in an ecological fitness cost in the absence of the stress, relative to individuals without the adaptation (Coustau et al., 2000; Heil, 2002). Such fitness costs can be allocation based, whereby limited plant resources are constitutively allocated to defense instead of to growth or reproduction (e.g., Herms and Mattson, 1992; Strauss et al., 2002). Alternatively, resistance mutations may directly affect enzyme-mediated reactions, e.g., via reduced catalytic capacity or substrate affinity, resulting in a subsequent fitness cost (Vila-Aiub et al., 2015). Some empirical evidence in support of this has been observed, for example costs of both constitutive and induced defense against herbivory have been documented (Strauss et al., 2002). Nevertheless, despite a sound theoretical background, such costs have remained difficult to conclusively demonstrate across taxa and disciplines (e.g., Laine, 2016). The rapid evolution of resistance to xenobiotics (e.g., fungicides, insecticides, herbicides) provides an exemplary model system with which to study such fitness trade-offs (Coustau et al., 2000; Vila-Aiub et al., 2011). In weeds, resistance to herbicides can evolve via mutations which alter expression of the herbicide target enzyme or reduce herbicide-target binding (target site resistance, TSR), or via changes in the rates of herbicide absorption, translocation, degradation or sequestration (non-target-site resistance, NTSR), (Powles and Yu, 2010). Within NTSR, enhanced metabolism of the herbicide molecule (EMR) is now widely reported as an important resistance mechanism (Yuan et al., 2007; Délye, 2013; Yu and Powles, 2014; Scarabel et al., 2015), and the extent to which this mechanism affects plant fitness is now being increasingly considered (Vila-Aiub et al., 2005a, 2009a; Keshtkar et al., 2017a,b).

Empirical support for fitness costs associated with herbicide resistance has been shown (Vila-Aiub et al., 2009b). A single resistance-endowing point-mutation at position 2078 of the acetyl-CoA carboxylase (ACCase) gene is associated with reduced ACCase activity, resulting in reduced growth rate and fecundity in some species (Menchari et al., 2007; Vila-Aiub et al., 2015). Similarly, a double point mutation in the 5-enolpyruvylshikimate-3-phosphate synthase (EPSPS) gene in *Eleusine indica* responsible for glyphosate resistance caused a considerable (50 – 85%) reduction in fecundity (Han et al., 2017). EPSPS gene amplification delayed seedling emergence and flowering, and caused reductions in competitive ability, seed number and weight of *Kochia scoparia* (Martin et al., 2017), and P450 mediated herbicide metabolism caused reduced growth and fecundity of *Lolium rigidum* (Vila-Aiub et al., 2005a, 2009a). Nevertheless, the broader conclusion arising from several decades of fitness research is that such costs are not ubiquitous, and instead can vary by organism, resistance mechanism, and even population (Tranel and Wright, 2002; Paris et al., 2008; Powles and Yu, 2010; Wan et al., 2017). Five different ALS resistance substitutions had little to no effect on acetolactate synthase (ALS) activity and plant growth rates in *Lolium rigidum* (Yu et al., 2010). Non-target-site resistance to ACCase herbicides in *Alopecurus myosuroides* had no effect on vegetative or reproductive fitness under competition with winter wheat (Keshtkar et al., 2017b), and no evidence was found for fitness costs in NTSR populations of *Apera spica-venti*, instead the NTSR populations had earlier germination and flowering times (Babineau et al., 2017).

One potential explanation is that costs may not be manifest under relatively benign experimental conditions. Rather, the costs of defense may be increasingly apparent under more stressful conditions, known as the defense-stress cost (DSC) hypothesis (Siemens et al., 2003). This may be the case for some instances of herbicide resistance, as nitrogen deficiency and competition with wheat exacerbated the fitness costs associated with resistance to photosystem-II inhibitors in *Brachypodium hybridum* (Frenkel et al., 2017). Similarly, whilst the number of viable offspring is generally taken as a reliable measure of fitness, resistance may cause more subtle alteration of life-history characteristics at other developmental stages (e.g., Délye et al., 2013). Although not affecting fitness directly, these could have considerable influence on realized plant fitness in the field via interaction with other management or environmental conditions (Colbach et al., 2016). Finally, many previous studies have been criticized for failure to account for differences in the genetic background of the R and S phenotypes used for study, with the potential to confound or mask observed fitness costs (Vila-Aiub et al., 2009a, 2011). As a result, it is important to ensure that tested populations differ only in the resistance trait through comparison of resistant and susceptible genotypes in a common or homogenized genetic background, that fitness is measured at multiple plant developmental stages, and across a range of environmental conditions such as weed-crop competition or abiotic stresses (Cousens and Fournier-Level, 2018).

In the UK, *Alopecurus myosuroides* is the principal weed species affecting cereal cropping, with evolved resistance to seven herbicide modes of action documented in this species (Heap, 2018). Resistance to the ALS sulfonylurea herbicides has led to rapid expansion of *A. myosuroides* infestations (Hicks et al., 2018), with enhanced herbicide metabolism being of particular concern in this species (Délye et al., 2011; Tétard-Jones et al., 2018). Both target-site and non-target-site mechanisms of resistance to ACCase herbicides have limited direct effects on reproductive fitness in this species, but cause specific alterations in germination and seedling establishment (Délye et al., 2013; Keshtkar et al., 2017a), see Table 1. However, to date no studies have evaluated the potential fitness costs of resistance to the Acetolactate-synthase (ALS) inhibiting herbicides in this species. In the current study, the presence of enhanced metabolism resistance (EMR) to a commercial formulation of the sulfonylurea (ALS) actives mesosulfuron and iodosulfuron, is investigated for its effect on plant life-history and fitness in *A. myosuroides*. Using phenotypes from a standardized genetic background, plant performance is assessed under conditions of intra- and inter-specific competition and nitrogen deprivation, at multiple stages of plant development.

**Table 1:** Previous studies into fitness costs associated with evolved herbicide resistance in *Alopecurus myosuroides*.

MOA	Mechanism	Finding	References
ACCase	TSR	Gly-2078 mutation caused a reduction in plant biomass, height, and seed production	Menchari et al., 2007
		Gly2078 mutation caused accelerated germination, Leu1781 mutation caused a delay in germination	Délye et al., 2013
		Costs of the Gly2078 mutation vary depending on the ‘S’ genetic background	Darmency et al., 2015
ACCase	NTSR	NTSR had no effect on vegetative biomass or tiller production	Keshtkar et al., 2017b
		NTSR caused reduced emergence of buried seeds	Keshtkar et al., 2017a


*The herbicide mode-of-action (MOA) for which resistance has been identified is listed, along with the resistance mechanism studied (TSR, target-site resistance; NTSR, non-target-site resistance).*

## Materials and Methods

### Generation of R and S Phenotypes

A UK field-collected population of blackgrass (*Alopecurus myosuroides*) with enhanced metabolism based herbicide resistance (EMR) to the ALS sulfonylurea actives mesosulfuron and iodosulfuron, but no known ALS target-site resistance, was used as a basis for this study. To generate appropriate seed populations for resistant (R) and susceptible (S) phenotypes so that fitness costs could be assessed in a common genetic background, two rounds of identification and interbreeding of R and S phenotypes was performed (see Vila-Aiub et al., 2011).

To produce the R phenotype, a set of 300 plants from this population were grown under glasshouse conditions to the five-leaf stage and sprayed with the herbicide ‘Atlantis’ (containing the active ingredients mesosulfuron and iodosulfuron). Plants were sprayed at the UK field-rate dose of 400 g ha^-1^ of the herbicide to identify resistant individuals. Leaf tissue was sampled from 48 surviving plants and screened for ALS target-site-resistance mutations using pyrosequencing (for details see Beffa et al., 2012; Hess et al., 2012). The presence of non-target-site-resistance was confirmed in these individuals using HPLC of leaf tissue inoculated for 12 h with C-14 radiolabelled mesosulfuron, to quantify enhanced metabolism as described in Collavo et al. (2013). Twenty plants with confirmed enhanced metabolism of the herbicide and with no ALS target-site mutations were chosen and were grown to maturity in a glasshouse and allowed to bulk-cross generating an F_1_ resistant seed population. Three hundred plants from this resistant F_1_ population were then grown to the five-leaf stage and sprayed with a 1200 g ha^-1^ dose of Atlantis. The 20 healthiest survivors were identified and bulk-crossed in a glasshouse, to generate the R seed population used in the current study.

To produce the S phenotype, 300 individuals from the source population were grown to the 5–6 tiller stage. These plants were vegetatively split to create three, single-tiller clones of each individual. Two clones from each plant were sprayed with a 240 g ha^-1^ dose of Atlantis, and 20 plants which were killed by this dose were selected. The unsprayed clones of these susceptible plants were grown to maturity in a glasshouse and allowed to cross-pollinate, creating an F_1_ susceptible seed population. Nine hundred plants of this F_1_ susceptible population were grown to the 5–6 tiller stage as previously and split into four single-tiller clones per individual. Three clones per plant were sprayed with a 120 g ha^-1^ dose of Atlantis, and 20 individuals for which all three sprayed clones died were identified. The unsprayed clones of these 20 plants were bulk-crossed as before to generate the S seed population used in the current study.

### Confirmation of R and S Phenotypes

A glasshouse dose-response experiment using the herbicide Atlantis was used to confirm that these R and S seed fractions, generated from a single seed population, represented different resistance phenotypes. Seeds from the second generation R and S fractions, plus the original field-collected population were pre-germinated in Petri dishes containing filter paper (Whatman N° 1) soaked with 2 g L^-1^ KNO_3_, and maintained within an incubator (Sanyo, MLR-350) for 6 days with a 17/11°C temperature cycle and a 14/10 h light/dark cycle. Germinated seedlings were sown individually into 6 cm pots containing a Kettering loam soil mixed with 2 kg m^-2^ Osmocote fertilizer and maintained in a glasshouse at approximately 16/10°C until the plants had reached the three-leaf stage. For herbicide application, pots were removed from the glasshouse and sprayed using a fixed track sprayer with a Teejet 110015VK nozzle mounted 50 cm above the plants. Boom speed was 0.33 m s^-1^, delivering herbicide at a volume of 212 L ha^-1^. Herbicide doses were varied according to the expected phenotype of the population, so that: plants from the S phenotype were sprayed at doses of 0, 12.5, 25, 50, 100, 200, 400, and 800 g ha^-1^. Plants from the field-collected baseline population were sprayed at 0, 50, 100, 200, 400, 800, 1600, and 3200 g ha^-1^, while plants from the R phenotype were sprayed at 0, 100, 200, 400, 800, 1600, 3200, and 6400 g ha^-1^. Fifteen replicate plants from each population were sprayed at each dose (*n* = 15). After spraying, pots were returned to the glasshouse for a further 3 weeks before visual assessment of the number of surviving plants per dose. The above-ground tissue from each plant was harvested and oven-dried at 80°C for 48 h before weighing to determine plant biomass.

Dose-response curves were fitted to the herbicide phenotyping data using the package ‘drc’ within R version 3.4.2. A range of 2- 3- and 4-parameter log logistic and Weibull type 1 and 2 models were compared using AIC, with a three-parameter log-logistic regression found to provide the best fit for biomass data, while a two-parameter binomial log logistic regression was optimal for assessment of the survival data. A post-hoc pairwise comparison of ED_50_ values for each model was used as a simple test to identify differences in resistance between R, S and baseline seed populations. Where ED_50_ values were significantly different, a ‘resistance index’ (RI) was calculated by dividing the higher ED_50_ by the lower to provide an approximate quantification of the relative difference in herbicide resistance between the seed populations.

### Experiment 1: Vegetative Establishment and Intraspecific Competition

In order to assay the performance of the ‘R’ and ‘S’ phenotypes under ecological gradients of inter- and intra-specific competition and nitrogen availability, we have used an outdoor, pot-based approach as adopted in other herbicide resistance fitness costs studies (Vila-Aiub et al., 2009a). This approach does not necessarily replicate true field conditions, but rather provides an optimal balance between exposure to ambient environmental conditions, whilst allowing accurate manipulation of environmental gradients.

**FIGURE 1:**
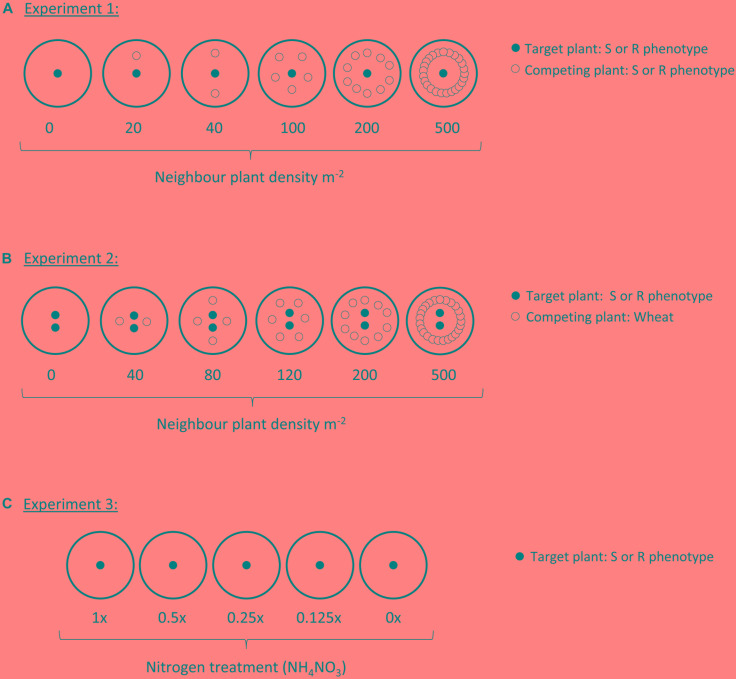
Diagram of planting designs within each of the three experiments. Each large circle represents a single pot, either filled with a Kettering loam soil (Experiments 1 and 2), or sand (Experiment 3). Small black-filled circles represent the target plant, always either an R or S blackgrass phenotype. Small hollow circles in Experiments 1 and 2 show the position of neighbor plants, either R or S phenotypes of blackgrass (Experiment 1), or wheat (Experiment 2).

A target-neighbor design was set up to evaluate establishment and early development of the two blackgrass phenotypes (resistant: R, and susceptible: S) under varying levels of intraspecific competition. Seeds of the two phenotypes were pre-germinated as previously. Pots (25 cm) were filled with a Kettering loam soil containing 2 kg m^-2^ osmocote fertilizer. ‘Target’ plants were established by sowing 120 pots with a single R seedling in the center, while a further 120 were sown with a single S seedling in the center. Further ‘neighbor’ blackgrass plants were then sown at different planting densities in a ring around each of these central plants, to provide varying levels of intraspecific competition. Numbers of neighbor plants were 0, 1, 2, 5, 10, and 25, representing competitor planting densities of 0, 20, 40, 100, 200, and 500 plants m^-2^. Sixty of the pots of each target phenotype were established with R neighbor plants, while the other half were established with S neighbor plants (see Figure 1A). In total, 10 replicate pots were established at each planting density for each combination of target and neighbor phenotypes (*n* = 10).

Pots were randomized and maintained outside, exposed to ambient environmental conditions at Harpenden (Hertfordshire, United Kingdom) during December – April. On seven occasions, target plants were non-destructively assessed for the number of tillers and the length of the longest tiller. For analysis, the dates of each non-destructive measurement were converted to thermal time, calculated as cumulative growing degree days above a base temperature of 1°C, as per Chauvel et al. (2000). At the end of April (155 days, ∼ 900 GDD) all pots were destructively harvested, separately collecting the aboveground tissue of the target plant and the combined aboveground tissue from all neighbor plants. Harvested tissue was oven dried at 80°C for 48 h before weighing to determine biomass.

Non-linear curve fitting approaches were explored to analyze these data, including fitting hyperbolic relationships as in previous studies (Vila-Aiub et al., 2009a). However, visual inspection of the models found that the fitted curves did not accurately fit the data. Instead, a generalized additive modeling approach was used. For the destructive biomass harvest, a model was fitted with target phenotype and neighbor phenotype as fixed effects, and with smoothed terms fitted for the effects of neighbor biomass, neighbor biomass × target phenotype, and neighbor biomass × neighbor phenotype. For the non-destructive measures of tiller length and number, generalized additive mixed-models were fitted, with target phenotype and neighbor phenotype as fixed effects, and with smoothed terms fitted for the effects of thermal time, thermal time × target phenotype, and thermal time × neighbor phenotype. Due to the repeated-measures nature of the data, a nested term for plant ID within thermal time was included as a random factor. To avoid model overfitting, neighbor number was not included as a fixed effect, but rather included as a further random term to account for differences in competitor density.

### Experiment 2: Reproductive Fitness Under Interspecific Competition

A further target-neighbor design was established to evaluate the reproductive productivity of the two blackgrass phenotypes when grown under interspecific competition with wheat. Twenty five centimeter pots containing Kettering loam soil were sown in a target-neighbor design similar to Experiment 1 (Figure 1B). In this case, neighbor plants were wheat of a variety commonly grown in the UK (JB Diego), at planting densities representing 0, 40, 80, 120, 200, and 500 competitor plants m^-2^. Pots were maintained outdoors under ambient conditions as previously over December to July, with supplementary watering provided by overhead sprinklers as necessary. As blackgrass is an obligate outcrossing species, two ‘target’ blackgrass plants were grown per pot to ensure cross-pollination and the production of viable seeds for estimation of reproductive fitness. At the onset of seed shedding, target plants were bagged with a micro-perforated plastic bag to collect the shed seed. Once the wheat seed-heads had matured, the aboveground material from all pots was destructively harvested, keeping the aboveground tissue of the target plants and the combined aboveground tissue from all neighbor plants separate. After harvest, the number and length of all blackgrass seed-heads per pot was evaluated as a measure of seed production (fecundity). The vegetative material (leaf and stem tissue) was then separated from the reproductive tissue (mature and immature seeds), before oven drying at 80°C for 48 h and weighing to determine vegetative and reproductive biomass. A simple measure of blackgrass productivity was calculated as the ratio of reproductive biomass: vegetative biomass, providing a measure of investment into reproductive tissue per gram of vegetative tissue. For all traits (vegetative biomass, reproductive biomass, reproductive productivity, maximum tiller length, seed head number, and total seed head length), the effect of wheat (neighbor) biomass, target phenotype, and their interaction were assessed using linear regression in R version 3.4.2.

### Experiment 3: Maintenance of Fitness Under Nitrogen Deprivation

A sand-culture experiment was used to identify any differences in life-history of the two blackgrass phenotypes at different levels of nitrogen availability. Seeds of each phenotype were pre-germinated within an incubator as in the previous experiments. Single seedlings were sown into the center of 20 cm pots containing a 1:1 mixture of pre-washed coarse and fine sand. Pots were arranged in a randomized block and maintained outside over March – August at Harpenden (Hertfordshire, United Kingdom). Each week, pots were fertilized with 500 ml of a Long-Ashton hydroponic nutrient solution. The ammonium nitrate (NH_4_NO_3_) concentration was varied to provide five nitrogen addition treatments, i.e., 1x, 0.5x, 0.25x, 0.125x, and 0x of the standard concentration of 0.05 g L^-1^ NH_4_NO_3_. Thirty pots of each blackgrass phenotype were treated with each level of nitrogen availability. To avoid nutrient build-up, pots were flushed with excess water and allowed to drain approximately 12 h before each nutrient addition. After 8 weeks, nutrient additions were doubled to twice per week, to avoid dehydration and to support greater plant biomass.

Tiller number and length of the longest tiller were measured non-destructively on five occasions over March – May. After 10 weeks growth, fifteen replicate plants of each blackgrass phenotype and nitrogen level were destructively harvested (*n* = 15). At this time, plants were large with approximately 30 tillers, but remained vegetative with no visible signs of floral initiation. Whole plants were carefully removed from pots, root systems washed in water, and leaf area measured using a Windias leaf area meter (Delta-t systems). Above- and below-ground tissues were separately dried at 80°C for 48 h and weighed to determine biomass. Root: shoot ratio was calculated from the below- and above-ground dry weights (g). Specific leaf area (SLA, ratio of leaf area: leaf weight) and leaf weight fraction (LWF, ratio of leaf weight: total plant weight) were also calculated as per Hunt et al. (2002). A further 15 plants of each phenotype × nitrogen dose (*n* = 15) were maintained in their respective fertilization treatments until reproductive maturity. Plants were allowed to openly cross-pollinate, before being individually bagged at the onset of seed shedding. At maturity, all plants were destructively harvested by removing all aboveground material. The length of the longest tiller, number of seed-heads, and combined length of all seed heads per plant were measured. Aboveground material was then separated into vegetative material (leaf and stem tissue) and reproductive tissue (mature and immature seeds), before oven drying at 80°C for 48 h and weighing. ‘Productivity’ was calculated as previously as the ratio of reproductive biomass: vegetative biomass.

Non-destructive tiller length and the log-transformed tiller number measurements were analyzed using a generalized additive mixed model approach as in Experiment 1. In this case, target phenotype, nitrogen treatment, and their interaction were included as fixed effects, with smoothed terms fitted for the effects of thermal time and the interaction between thermal time and target phenotype. A nested term for plant ID within thermal time was also included as a random factor due to the repeated-measures nature of the data. Vegetative and reproductive traits assessed during the two destructive harvests were analyzed using linear regression, assessing the fixed effects of plant phenotype, nitrogen treatment, and their interaction. All analyses were performed in R version 3.4.2.

## Results

### Confirmation of Resistance Phenotypes

**FIGURE 2:**
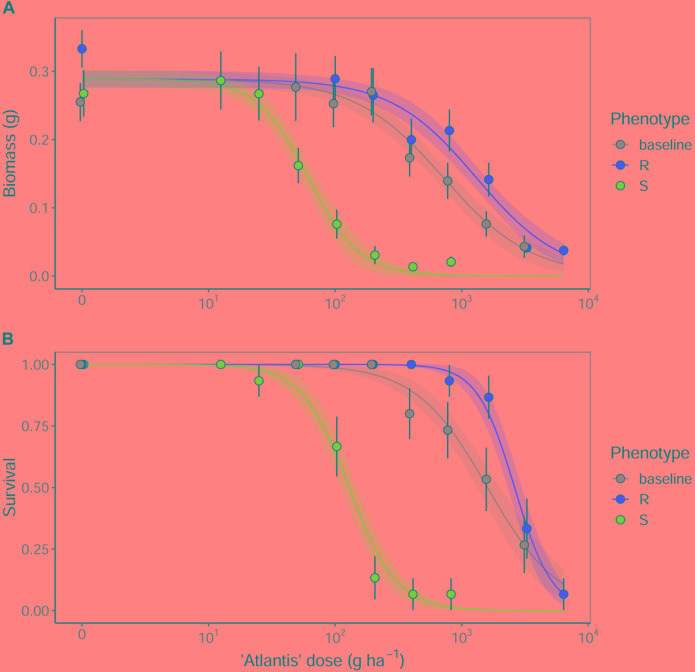
The fitted dose-response relationships for the models summarized in Table 2 for **(A)** aboveground biomass, and **(B)** proportional survival of R, S and baseline seed populations. Results are from a glasshouse dose-response experiment using the herbicide ‘Atlantis,’ containing the sulfonylurea active ingredients mesosulfuron and iodosulfuron. Solid lines show the fitted relationships, with shaded regions representing the 95% prediction interval. Points show the mean value of the data at each dose, with error bars showing ± 95% CI.

Analysis of the glasshouse herbicide phenotyping data confirmed a significant difference in the degree of herbicide susceptibility between the selected R and S seed populations to the sulfonylurea herbicide ‘Atlantis’ (containing the active ingredients mesosulfuron and iodosulfuron) (Figure 2). Significant differences were found between ED_50_ values of the different seed populations in both plant biomass and survival, with the resistant R phenotype having the highest ED_50_, the susceptible S phenotype the lowest, and with the baseline population showing an intermediate resistance between these (Table 2). The Resistance Index (RI) of the R seed phenotype was approximately 20x higher than the S fraction, consistent across both the biomass and survival data. These results confirm that the R and S seed fractions represent contrasting resistance phenotypes.

**Table 2:** Confirmation of R and S phenotypes to the herbicide Atlantis (active ingredients mesosulfuron + iodosulfuron).

Comparison	ED_50_, g ha^-1^ (*SE*)	RI	*t*-value	*P*-value
**Biomass**		
Baseline	717.2 (*148.1*)	11.7	4.469	<0.001^∗∗∗^
R	1302.2 (*267.9*)	21.3	4.660	<0.001^∗∗∗^
S	61.2 (*9.1*)	–	–	–
**Survival**		
Baseline	1600.0 (*327.4*)	12.4	7.460	<0.001^∗∗∗^
R	2619.6 (*333.3*)	20.3	4.485	<0.001^∗∗∗^
S	129.1 (*18.3*)	–	–	–


*Aboveground dry-weight data (biomass) was analyzed using a three-parameter log-logistic regression model, while plant survival was analyzed using a binomial two-parameter model. The Resistance Index (RI) has been calculated for the Baseline and resistant (R) seed populations relative to the susceptible (S) seed population, as the fold-difference between the compared ED_*50*_ values. Significance (*t*- and *P*-values) shown are results of *post hoc* pairwise comparison of ED_*50*_ values for the Baseline and R populations relative to the ED_*50*_ of the S population using a *Z*-test (asterisks denote significance at: ^∗∗∗^*P* ≤ 0.001).*

### Vegetative Establishment and Intraspecific Competition

**FIGURE 3:**
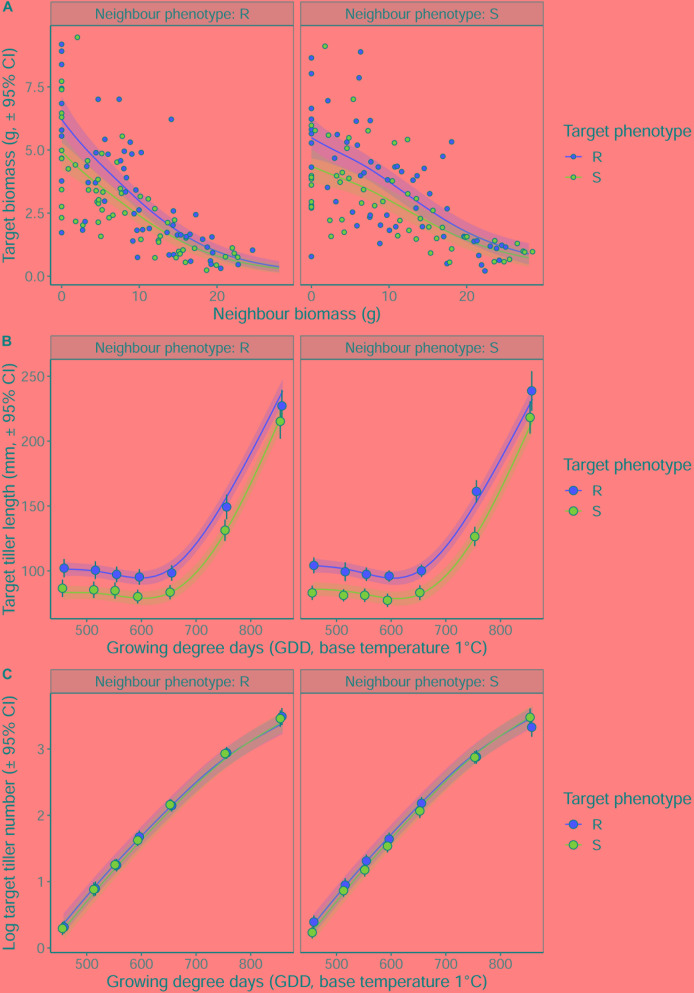
The impact of interspecific competition from neighboring R or S blackgrass plants on vegetative development of target blackgrass plants. **(A)** Target plant biomass after 155 days. **(B)** Target plant maximum tiller length. **(C)** Log-transformed target plant tiller number. Solid lines represent fitted data from generalized additive mixed models with shaded regions representing the 95% prediction interval.

Averaged over all competition treatments and time-points, the R phenotype plants had significantly greater tiller lengths, 17% longer than their S counterparts (Figure 3). The greater tiller length of the R phenotype became less pronounced following rapid tiller elongation of both phenotypes at ∼650 growing degree days, resulting in a significant phenotype × thermal time interaction (Table 3). Plants of the R phenotype also had significantly greater biomass than S blackgrass under low levels of competition (Figure 3). Increased competitor biomass led to a significant reduction in biomass of both blackgrass phenotypes, though competition with neighbor plants of the R phenotype caused a more rapid reduction in target biomass (Figure 3), observed as a significant neighbor biomass × neighbor phenotype interaction (Table 3). This demonstrates that R neighbor plants produced a greater competitive effect per gram of biomass than the S neighbor plants over this time. Tiller number increased significantly over the course of the experiment, but no significant differences were found between the two phenotypes (Table 3).

**Table 3:** Analysis of R and S blackgrass phenotypes during vegetative growth at different levels of intraspecific competition.

Term	DF	*F*	*P*
**Tiller number**		
Target phenotype	1.000	1.533	0.216
Neighbor phenotype	1.000	0.644	0.422
Growing degree days	3.863	1048.559	< 0.001***
Growing degree days × Target phenotype	1.527	3.398	0.1064
Growing degree days × Neighbor phenotype	1.000	3.075	0.0797
**Tiller length**		
Target phenotype	1.000	34.944	< 0.001 ***
Neighbor phenotype	1.000	0.068	0.794
Growing degree days	3.964	621.536	< 0.001 ***
Growing degree days × Target phenotype	3.535	2.835	0.0151*
Growing degree days × Neighbor phenotype	1.180	2.649	0.1246
**Vegetative biomass**		
Target phenotype	1.000	10.419	0.001**
Neighbor phenotype	1.000	9.941	0.002*
Neighbor biomass	2.330	18.97	< 0.001 ***
Neighbor biomass × Target phenotype	1.000	0.02	0.889
Neighbor biomass × Neighbor phenotype	1.000	12.57	< 0.001 ***


*Tiller number and tiller length were assessed non-destructively on plants during establishment and vegetative growth. Vegetative biomass was assessed following a destructive harvest 155 days after planting. Significance of the effects of target plant phenotype, neighbor plant phenotype, and either thermal time or neighbor biomass were assessed using a generalized additive mixed modeling approach. The degrees of freedom (DF), F-statistic (F) and associated *P*-value (P) are shown, with asterisks denoting significance at: ^∗^*P* ≤ 0.05, ^∗∗^*P* ≤ 0.01, ^∗∗∗^*P* ≤ 0.001).*

### Maintenance of Fitness Under Interspecific Competition

**FIGURE 4:**
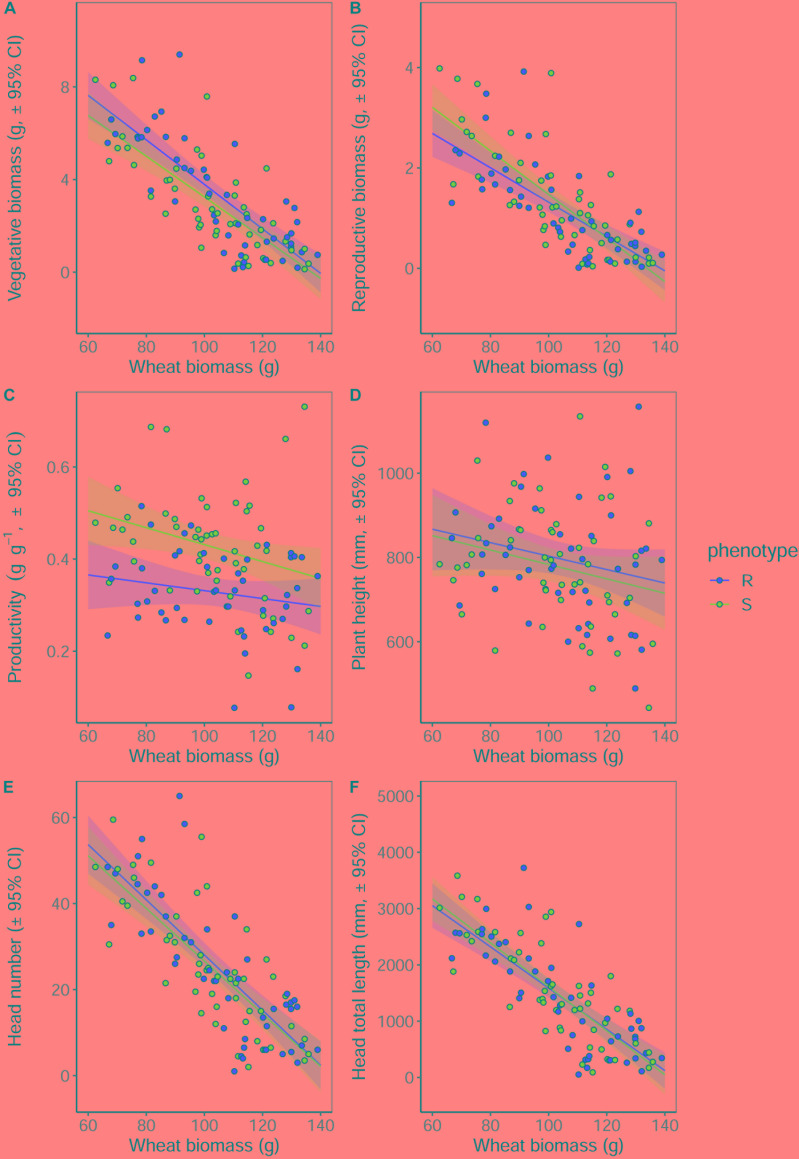
Growth and reproductive potential of blackgrass R and S phenotypes under competition with wheat, measured destructively at reproductive maturity. **(A)** Aboveground vegetative biomass (leaf and stem tissue). **(B)** Reproductive biomass (seeds and seed heads). **(C)** The ratio of reproductive biomass: vegetative biomass, providing an estimate of investment into reproductive tissue per gram of vegetative tissue. **(D)** Maximum tiller height. **(E)** Number of seed heads. **(F)** Total combined length of all seed heads. Solid lines show the linear fitted relationship, with shaded regions representing the 95% prediction interval.

Competition with wheat was found to significantly reduce blackgrass growth and reproductive potential, with little difference between blackgrass phenotypes. Increased wheat biomass was found to significantly reduce all of the measured blackgrass traits (Table 4). Per gram of wheat biomass, blackgrass biomass was reduced by 0.13 g, and total seed head length reduced by 36.6 mm (Figure 4). No differences between R and S phenotypes were observed in total biomass, seed-head length, plant height, and seed head number. However, the S phenotype allocated a significantly greater proportion of resources to reproductive tissue than the R phenotype (Table 4 and Figure 4). This is notable, as it indicates that S dedicate a proportionally greater allocation of resources to reproduction.

**Table 4:** Analysis of the effect of competition with neighbor wheat plants on the growth and reproductive potential of R and S blackgrass target plants.

Term	DF	SSq	*F*	*P*
**Vegetative biomass**		
Wheat biomass	1	314.609	150.915	< 0.001***
Target phenotype	1	6.492	3.114	0.081
Wheat biomass × Target phenotype	1	0.612	0.294	0.589
**Reproductive biomass**		
Wheat biomass	1	56.683	127.038	< 0.001***
Target phenotype	1	0.346	0.777	0.380
Wheat biomass × Target phenotype	1	0.785	1.760	0.188
**Reproductive productivity**		
Wheat biomass	1	0.082	7.224	0.008**
Target phenotype	1	0.229	20.095	< 0.001***
Wheat biomass × Target phenotype	1	0.009	0.798	0.374
**Maximum height**		
Wheat biomass	1	98.544	5.039	0.027*
Target phenotype	1	10078	0.515	0.475
Wheat biomass × Target phenotype	1	119	0.006	0.938
**Seed-head number**		
Wheat biomass	1	14738.8	157.166	< 0.001***
Target phenotype	1	41.9	0.447	0.506
Wheat biomass × Target phenotype	1	8.1	0.086	0.770
**Total seed-head length**		
Wheat biomass	1	53845108	168.420	< 0.001***
Target phenotype	1	4753	0.015	0.9032
Wheat biomass × Target phenotype	1	53238	0.167	0.6841


*All measurements were taken destructively at reproductive maturity. Reproductive productivity represents the ratio of reproductive biomass (seed + seed-heads) : vegetative biomass (leaf + stem tissue), providing an estimate of investment into reproductive tissue per gram of vegetative tissue. Significance values following linear regression analysis are reported. The degrees of freedom (DF), sums-of-squares (SSq), F-statistic (F) and associated *P*-value (P) are shown, with asterisks denoting significance at: ^∗^*P* ≤ 0.05, ^∗∗^*P* ≤ 0.01, ^∗∗∗^*P* ≤ 0.001).*

### Vegetative Establishment Under Nitrogen Deprivation

**FIGURE 5:**
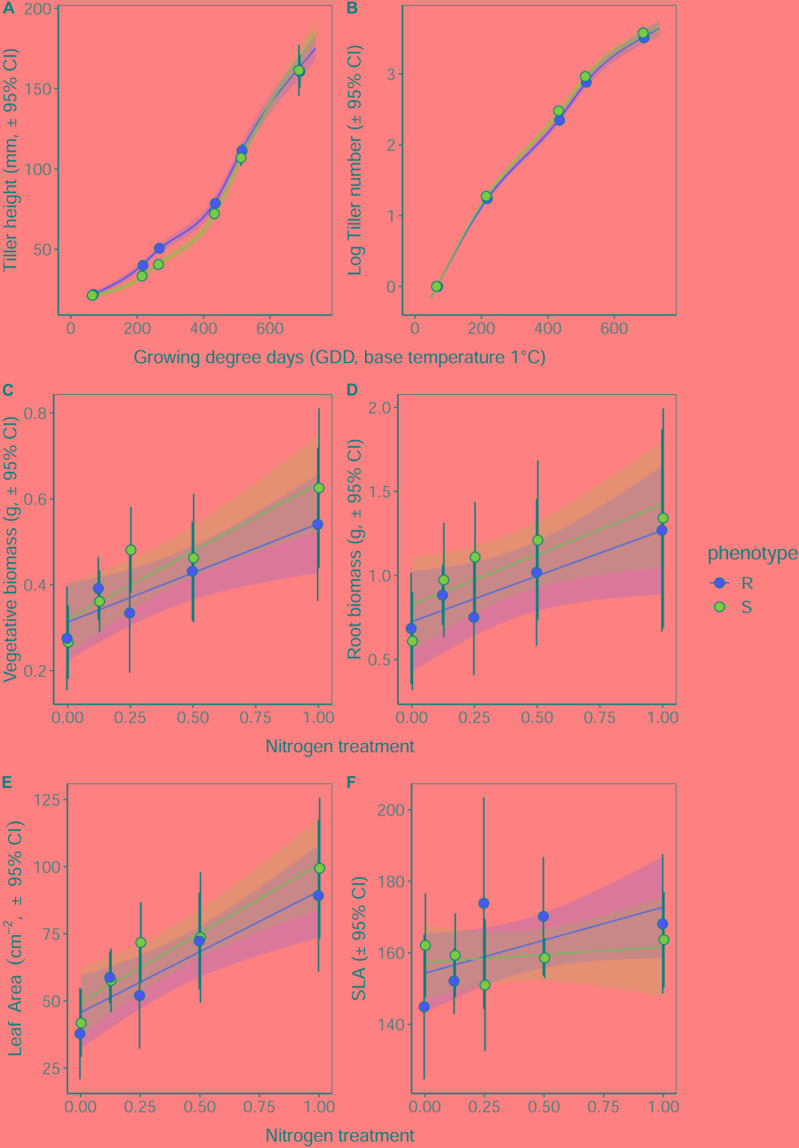
Effect of nitrogen fertilization on vegetative development and morphology of R and S blackgrass phenotypes. **(A)** Tiller length and **(B)** number of tillers, assessed non-destructively during plant establishment and vegetative growth. **(C)** Aboveground biomass, **(D)** root biomass, **(E)** total leaf area, and **(F)** specific leaf area were assessed destructively after 72 days growth. Solid lines show the fitted relationship, with shaded regions representing the 95% prediction interval.

As in the previous competition experiment, plants of the R phenotype grown in sand-culture were found to have a significantly greater tiller length during early development, with this effect becoming less pronounced over time (Table 5 and Figure 5). Destructive harvest of plants after 72 days, however, identified no further significant effects of phenotype on plant growth characteristics, nor any interaction between phenotype and nitrogen treatment (Table 6). The nitrogen treatment did affect plant development, with higher nitrogen fertilization resulting in greater above- and below-ground biomass accumulation of both blackgrass phenotypes (Table 6). No further effects of nitrogen treatment were observed on other aspects of plant morphology, measured as the root: shoot ratio, SLA and LWF.

**Table 5:** Analysis of vegetative development of R and S blackgrass phenotypes grown in sand-culture at different levels of nitrogen fertilization.

Term	DF	*F*	*P*
**Tiller length**		
Phenotype	1	7.103	0.008**
Nitrogen	1	0.279	0.597
Phenotype × Nitrogen	1	0.552	0.457
Growing degree days	4.88	404.12	< 0.001 ***
Growing degree days × Phenotype	3.288	12.52	< 0.001***
**Log Tiller number**		
Phenotype	1	3.589	0.058
Nitrogen	1	0.877	0.349
Phenotype × Nitrogen	1	0.952	0.329
Growing degree days	3.969	1890.290	< 0.001***
Growing degree days × Phenotype	2.607	3.392	0.015*


*Tiller number and tiller length were assessed non-destructively on plants during the course of establishment and vegetative growth. Significance of the effect of plant phenotype, nitrogen fertilization, thermal time, and their interactions was assessed using a generalized additive mixed modeling approach. The degrees of freedom (DF), F-statistic (F) and associated *P*-value (P) are shown, with asterisks denoting significance at: ^∗^*P* ≤ 0.05, ^∗∗^*P*≤ 0.01, ^∗∗∗^*P* ≤ 0.001).*

**Table 6:** Effect of nitrogen fertilization under sand-culture conditions on the vegetative growth and morphology of R and S blackgrass phenotypes.

Term	DF	Sum-Sq	*F*	*P*
**Leaf weight**		
Phenotype	1	0.030	0.758	0.387
Nitrogen	1	0.885	22.756	< 0.001 ***
Phenotype^∗^Nitrogen	1	0.022	0.569	0.4529
**Root weight**		
Phenotype	1	0.277	0.642	0.425
Nitrogen	1	3.849	8.927	< 0.001 ***
Phenotype^∗^Nitrogen	1	0.008	0.019	0.453
**Leaf area**		
Phenotype	1	644	0.723	0.398
Nitrogen	1	27651	31.071	< 0.001 ***
Phenotype^∗^Nitrogen	1	113	0.127	0.722
**Specific leaf area (SLA)**		
Phenotype	1	170	0.287	0.594
Nitrogen	1	1477	2.485	0.119
Phenotype^∗^Nitrogen	1	590	0.993	0.322
**Leaf weight fraction (LWF)**		
Phenotype	1	0.00002	0.002	0.961
Nitrogen	1	0.00148	0.189	0.665
Phenotype^∗^Nitrogen	1	0.00109	0.140	0.710
**Root:Shoot ratio**		
Phenotype	1	0.006	0.008	0.930
Nitrogen	1	0.356	0.423	0.517
Phenotype^∗^Nitrogen	1	0.060	0.071	0.791


*All measurements were assessed destructively after 72 days growth. Specific leaf area (SLA) was calculated as the ratio of leaf area: leaf dry weight. Leaf weight fraction (LWF) is the ratio of leaf dry weight: total plant dry weight. Significance values following linear regression analysis are reported. The degrees of freedom (DF), sums-of-squares (SSq), F-statistic (F) and associated *P*-value (P) are shown, with asterisks denoting significance at: ^∗∗∗^*P* ≤ 0.001).*

### Maintenance of Fitness Under Nitrogen Deprivation

**FIGURE 6:**
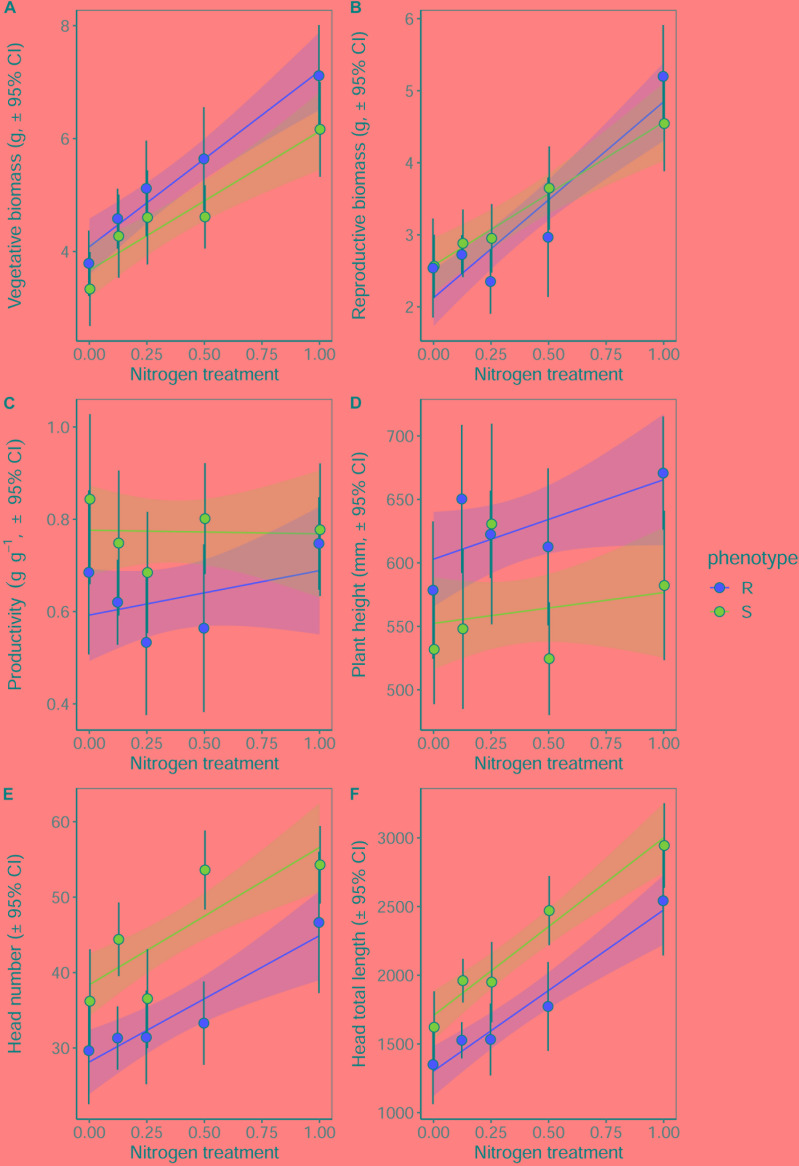
Growth and reproductive potential of R and S blackgrass phenotypes grown in sand-culture at five levels of nitrogen fertilization. Measurements were taken destructively at reproductive maturity. **(A)** Aboveground vegetative biomass (leaf and stem tissue). **(B)** Reproductive biomass (seeds and seed heads). **(C)** The ratio of reproductive biomass : vegetative biomass, providing an estimate of investment into reproductive tissue per gram of vegetative tissue. **(D)** Maximum tiller height. **(E)** Number of seed heads. **(F)** Total combined length of all seed heads. Solid lines show the linear fitted relationship, with shaded regions representing the 95% prediction interval.

Significant effects of both plant phenotype and nitrogen treatment were observed in plants at reproductive maturity, although no interactions were observed between these terms (Table 7). As at earlier stages of development, the maximum tiller length (plant height) was significantly greater for plants of the R phenotype, and these plants also had a significantly greater aboveground vegetative biomass (Figure 6). No difference was observed between the phenotypes in total reproductive biomass (combined weight of immature and mature seeds), however, consistent with results in competition with wheat, the ratio of reproductive: vegetative biomass was significantly greater in the S phenotype (Table 7 and Figure 6). Under these sand-culture conditions, the S phenotype also produced a significantly greater number of seed-heads, and total seed-head length (Table 7 and Figure 6). Nitrogen treatment had a significant effect on both vegetative and reproductive biomass, number of seed-heads, and seed head length (Table 7). In all cases, greater nitrogen doses were associated with greater values for these traits (Figure 6).

**Table 7:** Effect of nitrogen fertilization under sand-culture conditions on the reproductive potential of R and S blackgrass phenotypes.

Term	DF	Sum-Sq	*F*	*P*
***Total biomass***		
Phenotype	1	8.33	2.243	0.137
Nitrogen	1	466.38	125.642	< 0.001 ***
Phenotype^∗^Nitrogen	1	8.41	2.264	0.135
**Vegetative biomass**		
Phenotype	1	15.520	7.541	0.007**
Nitrogen	1	137.501	66.807	< 0.001***
Phenotype^∗^Nitrogen	1	1.854	0.901	0.344
**Reproductive biomass**		
Phenotype	1	1.111	0.871	0.352
Nitrogen	1	97.413	76.368	< 0.001***
Phenotype^∗^Nitrogen	1	2.364	1.853	0.176
**Reproductive productivity**		
Phenotype	1	0.7458	8.976	0.003**
Nitrogen	1	0.0305	0.367	0.546
Phenotype^∗^Nitrogen	1	0.0479	0.576	0.449
**Height**		
Phenotype	1	149452	12.916	< 0.001***
Nitrogen	1	34249	2.960	0.088
Phenotype^∗^Nitrogen	1	6507	0.562	0.455
**Head number**		
Phenotype	1	4121.3	27.194	< 0.001***
Nitrogen	1	5306.5	35.015	< 0.001***
Phenotype^∗^Nitrogen	1	9.2	0.061	0.806
**Total head length**		
Phenotype	1	7161323	25.739	< 0.001***
Nitrogen	1	26577482	95.522	< 0.001***
Phenotype^∗^Nitrogen	1	63606	0.229	0.633


*All measurements were assessed destructively when plants had reached reproductive maturity. Reproductive productivity represents the ratio of reproductive biomass (seed + seed-heads): vegetative biomass (leaf + stem tissue), providing an estimate of investment into reproductive tissue per gram of vegetative tissue. Significance values following linear regression analysis are reported. The degrees of freedom (DF), sums-of-squares (SSq), F-statistic (F) and associated *P*-value (P) are shown, with asterisks denoting significance at: ^∗∗^*P* ≤ 0.01, ^∗∗∗^*P* ≤ 0.001).*

## Discussion

Whilst our results did not reveal a consistent reproductive fitness cost associated with NTSR conveyed by enhanced metabolism (EMR) in *Alopecurus myosuroides,* we did observe a significant reduction in seed production potential of the R phenotype in the nutrient deprivation study. Individual R plants exhibited a 27% reduction in the mean per plant number of seed heads and an associated 23% reduction in mean total seed-head length. These measures of reproductive output are a viable proxy for fecundity in this species, for which other measures of total seed production are impractical (Keshtkar et al., 2017b). However, these reductions in seed-head numbers of R plants compared to S were not observed during interspecific competition with wheat. These contrasting results likely arise from the different growth media and planting dates and may indicate that fitness costs are accentuated when resources are limited by shorter growing seasons and/or more limited nutrient availability (see Vila-Aiub et al., 2009b). As these experiments were pot-based and conducted over a single year, we cannot preclude that other fitness costs could be manifest in the field under exposure to other annual climatic conditions. Similarly, the ‘R’ and ‘S’ phenotypes studied were derived from a single field collected population, and as such, fitness costs might be expected to differ in other populations if NTSR has evolved with a different genetic architecture or in a different genetic background. However, previous assessment of costs associated with NTSR to the acetyl-CoA carboxylase (ACCase) herbicides in *A. myosuroides* also found no evidence of reduced fecundity (Keshtkar et al., 2017b), suggesting that a severe reproductive fitness cost associated with NTSR is not present in this species.

Studies of NTSR mechanisms in other species have reported reductions in reproductive fitness. NTSR endowed by overexpression of cytochrome P-450 enzymes was shown to cause a 23% reduction in fecundity in *Lolium rigidum* (Vila-Aiub et al., 2009a). Similarly, NTSR to glyphosate caused by reduced translocation also resulted in a small reduction in fecundity of *Lolium rigidum* (Pedersen et al., 2007), and populations of *Avena fatua* with NTSR to multiple herbicide actives produced fewer seeds than an equivalent herbicide susceptible population (Lehnhoff et al., 2013b). Nevertheless, a growing body of evidence now indicates that the presence of NTSR is more often typified by an absence of resistance costs. For example, no evidence of reproductive fitness costs was found in multi-herbicide resistant populations of *Apera spica-venti* (Babineau et al., 2017), or in paraquat resistant *Hordeum leporinum* (Purba et al., 1996). No costs of NTSR on vegetative growth of *Avena fatua* were found under varying levels of competition or nutrient availability (Lehnhoff et al., 2013a), and variation in seed production was not consistently different from all susceptible populations (Lehnhoff et al., 2013b). Populations of *A. myosuroides* with enhanced metabolism-based resistance to Acetyl-CoA carboxylase (ACCase) herbicides had no observed reduction in fecundity (Keshtkar et al., 2017b), and glyphosate resistant lines of *Ipomoea purpurea* produced similar numbers of seed to susceptible lines in the absence of herbicide, suggesting no direct reproductive fitness cost (Debban et al., 2015). Therefore, while NTSR can result in significant reproductive fitness costs, this is clearly not a ubiquitous response across all resistance cases or species, and results such as the current study demonstrating no consistent reproductive costs are now growing more common.

One explanation for the absence of costs is that compensatory evolution might occur, such that any initial reduction in fitness will be rapidly recovered through the selection of compensatory mutations and/or the expression of resistance mutations in favorable genetic backgrounds (Paris et al., 2008; Darmency et al., 2015). This process has been confirmed as an important mechanism to allow recovery of fitness costs associated with antibiotic (Billal et al., 2011; Brandis et al., 2012) and insecticide resistance (Pischedda and Chippindale, 2005), and annual plants with extensive variation in fitness-related life history traits such as *A. myosuroides* are expected to have a considerable potential for rapid compensatory evolution and recovery of costs (Wan et al., 2017). Although study of this process in herbicide resistant plants remains scarce, Darmency et al. (2015) demonstrate some evidence for compensation of costs associated with an ACCase target-site resistance mutation in *A. myosuroides,* suggesting that compensatory evolution can occur in this species. The magnitude of any expected fitness cost may further depend on the origin or genetic architecture of the resistance allele(s) which have been selected, for example studies in the model plant species *Arabidopsis thaliana* identified that pathogen resistance conveyed by insertion–deletion (indel) polymorphisms have substantially higher costs than resistance genes with alternative R and S alleles (Laine, 2016; MacQueen et al., 2016). In practice therefore, reproductive fitness costs associated with NTSR could be specific to individual species, populations, or mutations, highlighting the difficulty in conclusively demonstrating such costs.

Rather than revealing substantial reductions in fecundity, emerging evidence suggests that resistance more often causes subtle alterations in patterns of life-history and resource allocation (e.g., Vila-Aiub et al., 2005a; Délye et al., 2013; Van Etten et al., 2016; Bravo et al., 2017), with the current study highlighting some consistent pleiotropic effects of NTSR on plant development during both vegetative establishment and at reproductive maturity. Plants of the R phenotype had consistently longer tillers during early development and were more competitive under intraspecific competition than S counterparts. These results are interesting in that they demonstrate a potential positive consequence of resistance on plant development. Such results are not unprecedented, for example an ACCase resistance allele in *Setaria italica* caused more vigorous growth, earlier flowering and greater seed production than a wild-type (Wang et al., 2010), and NTSR caused more rapid development and flowering in populations of *Apera spica-venti* (Babineau et al., 2017). An alteration in reproductive productivity was also observed, with susceptible plants allocating a greater proportion of biomass to reproductive, rather than vegetative, tissues at maturity. These findings are echoed in a study of ALS resistance in *Echinochloa crus-galli*, where no differences in total or vegetative biomass were found, but susceptible plants maintained a greater proportional investment to reproductive biomass per gram of total plant biomass (Panozzo et al., 2017). Other effects of NTSR on resource allocation have been described, for example Vila-Aiub et al. (2005a) demonstrate reduced growth rates and vegetative development in an NTSR population of *Lolium rigidum*, despite no overall effects on fecundity. Our results suggest a subtle alteration of plant resource partitioning associated with enhanced metabolism-based NTSR in *A. myosuroides*, although the mechanistic basis for this remains unknown.

Taken independently, variation in developmental traits does not directly impact reproductive fitness and so these differences cannot strictly be defined as costs of resistance (Cousens and Fournier-Level, 2018). However, the importance of pleiotropic differences in life history traits between R and S plants should not be dismissed, as these traits may interact with environmental and management variability to result in environmentally mediated differences in fitness. For example, despite finding no differences in fecundity associated with glyphosate resistance in *Ipomoea purpurea* (Debban et al., 2015), small reductions in seed viability, root growth, and aboveground shoot establishment in R phenotypes of this species are expected to cumulatively result in reduced fitness in novel or stressful environments (Van Etten et al., 2016). Alterations in seed dormancy associated with ACCase target-site mutations have been reported in populations of *A. myosuroides* (Délye et al., 2013), and *Lolium rigidum* (Vila-Aiub et al., 2005b). Whilst not directly impacting plant growth or fecundity, it has been suggested that the resultant effects on timing of germination could be exploited by careful timing of management interventions to select against the R phenotypes (Colbach et al., 2016). Nevertheless, whilst life-history analysis in the current study has revealed significant pleiotropic effects of NTSR on resource allocation, it is unlikely that the observed differences represent traits which could be exploited through alteration of agronomic practices.

Where fitness costs are small, considerable experimental ‘power’ is needed to detect those costs, with one recent estimate suggesting that differences of up to 25% might be being missed due to the large amount of ‘background’ variation in plant-to-plant fitness against which R and S costs are being measured (Cousens and Fournier-Level, 2018). To counteract this and to minimize false positives or negatives, it is necessary to minimize genetic background differences between the R and S phenotypes that are being compared (Vila-Aiub et al., 2011). However, the experimentally intensive process of creating genetically controlled R and S lines often necessitates assessment of just a small number of R and S genotypes, potentially missing differential effects of independently evolved R genotypes or interactions across multiple S genetic backgrounds. Where the genetic basis of resistance is known, a powerful technique is to monitor changes in the frequency of resistance alleles over time in un-sprayed field populations (Vila-Aiub et al., 2011), however, this is unsuitable for polygenic traits such as NTSR where the mutational basis of resistance remains unknown. Controlling for differences in genetic background, and providing sufficient replication for statistical power has required that the current study focus in considerable detail on NTSR from a single field collected population. Whilst minimizing false positive or negative results, if the genetic architecture for NTSR varies in other populations, effects on life-history or fitness might also be manifest differently. Instead, a novel approach for future herbicide resistance fitness studies would be to draw on evolutionary quantitative genetic analyses developed within other ecological and resistance disciplines (Juenger and Bergelson, 2000; Agrawal et al., 2002; Klerks et al., 2011). These methods directly measure the underlying genetic correlations between resistance and fitness traits and can be directly examined from pedigreed lines representing a broad range of independently evolved R and S genotypes, even when the underlying genetic basis of resistance is unknown (Wilson et al., 2010; de Villemereuil, 2018).

## Conclusion

Our results are in general agreement with the emerging consensus that direct, measurable reproductive costs of herbicide resistance are considerably smaller, or less frequent, than anticipated from theoretical predictions (Tranel and Wright, 2002; Powles and Yu, 2010; Wan et al., 2017). Whether this is due to rapid compensatory evolution, lack of experimental power or selection for mutations with little or no cost, remains a topic for further study. However, to more comprehensively understand the effects of resistance (particularly polygenic NTSR) on plant life-history, we advocate a change in approach from phenotypic comparison of R and S lines, to a more holistic quantitative genetics approach, examining genetic correlations amongst pedigreed lines from a range of R and S backgrounds.

## Data Availability

The datasets generated for this study are available on request to the corresponding author.

## Author Contributions

CK collected and segregated the seed populations under the supervision of PN and RB. DC and PN conceived and designed the fitness assays. DC, LC, and RH conducted all dose-response and plant growth experiments at Rothamsted Research (United Kingdom). RB conducted all metabolism analyses at BayerAG. DC statistically analyzed the data. DC and PN wrote the manuscript with contribution from all authors.

## Conflict of Interest Statement

CK contributed as part of a Ph.D. studentship co-funded by Bayer CropScience, supervised by RB and PN. The remaining authors declare that the research was conducted in the absence of any commercial or financial relationships that could be construed as a potential conflict of interest.
